# Insights into the expansion of Oropouche virus in Brazil: epidemiological and environmental aspects

**DOI:** 10.3389/ebm.2025.10647

**Published:** 2025-07-24

**Authors:** Igor Pereira Godinho, Ítalo Faria Dória, Victor de Melo Rocha, Bárbara Alves Miranda, Lucas Fernandes Chamhum Salomão, Brunello Stancioli, Adriana Alves Oliveira Paim, Jordana G. Alves Coelho dos Reis, Samille Henriques Pereira, Flávio Guimarães da Fonseca

**Affiliations:** ^1^ Laboratório de Virologia Básica e Aplicada, Departamento de Microbiologia, Universidade Federal de Minas Gerais, Belo Horizonte, Brazil; ^2^ Faculdade de Direito, Universidade Federal de Minas Gerais, Belo Horizonte, Brazil

**Keywords:** *Orthobunyavirus oropoucheense*, Oropouche fever, *Culicoides paraensis*, epidemiology, climate change, environmental impacts

## Abstract

The Oropouche virus (OROV), an arbovirus transmitted primarily by the *Culicoides paraensis* midge, has caused significant outbreaks in the Americas, especially in the Amazon region. The virus’s spread is closely linked to a combination of environmental, climatic, and ecological factors. These include deforestation, urbanization, and changes in rainfall patterns, which influence the proliferation of vectors, and, consequently, increase the chances of mutations and reassortment events to occur. In 2024 and 2025, the number of OROV cases increased significantly, with outbreaks extending beyond the traditionally endemic Amazon region, highlighting the growing geographic expansion of the disease throughout Brazil. Despite its growing dispersion, diagnostic and therapeutic tools for OROV remain limited. Current diagnostic strategies rely almost exclusively on molecular detection methods, and there are no vaccines for effective prevention. Additionally, immunological responses to OROV infection are not fully understood, and further studies are needed. The ecological dynamics influencing OROV transmission, particularly the role of environmental changes in shaping vector populations, highlight the need for more integrated surveillance and control strategies. The ongoing expansion of OROV outside its traditional hotspots may be indicative of broader environmental shifts that facilitate viral spread. Therefore, continuous monitoring of both environmental and epidemiological data is crucial to understanding and mitigating the impact of OROV in the future. Collaborative efforts among researchers, policymakers, and local communities will be essential to prevent further outbreaks and minimize the health burden caused by OROV. This review summarizes important and up-to-date data information to the ongoing epidemic of Oropouche fever, focusing on topics that are particularly important to Public Health.

## Impact statement

Climate change, environmental alterations due to anthropogenic activity, and human mobility have significantly altered the occurrence and dispersion of infectious diseases. This is particularly relevant when the disease is transmitted by arthropod vectors and/or has sylvatic hosts. Oropouche fever, caused by the Oropouche virus (OROV - Orthobunyavirus oropoucheense) is an iconic example of this new pattern of disease transmission. Since the virus description, in the 1950s, the OROV has remained restricted to equatorial forests, especially in the Amazon basin, where its main mosquito vector (Culicoides paraensis) is found. However, in the last 3 years, OROV cases have increased, and the virus is now dispersed to Brazil's states outside the Amazon region, posing a significant threat to public health resources. This review focuses on epidemiological characteristics of oropouche fever in Brazil and possible environmental aspects underlying the unprecedented OROV dispersion within the country. 

## Introduction

The Oropouche virus (OROV - *Orthobunyavirus oropoucheense*) is an arthropod-borne virus (arbovirus) belonging to the *Peribunyaviridae* family, *Orthobunyavirus* genus, and classified within the Simbu serogroup. Viruses in this group are known for their broad host range, vector-mediated transmission, and potential for reassortment events that contribute to genetic diversity. In 2024, OROV gained prominence due to a significant increase in reported cases across the Americas [1, 2].

The OROV was first isolated in 1955 from blood samples of a forestry worker in the Vega de Oropouche community, Trinidad and Tobago, a country in the Caribbean region of Central America [3]. This early identification marked the beginning of research on the virus, which was initially linked to a zoonotic transmission cycle involving both wild mammals and arthropod vectors. In 1960, the virus was detected in Brazil for the first time, isolated from a sloth (*Bradypus tridactylus*) near the construction site of the Belém-Brasília highway, state of Pará. In the same area, *Ochlerotatus serratus* mosquitoes were found to be infected with OROV [4]. Between 1960 and 1980, OROV was responsible for several epidemics in the state of Pará, with approximately 11,000 people infected. These epidemics affected different mesoregions - including metropolitan Belém - except for the southwestern part of the state. It was only from the 1980s onwards that cases of OROV infection began to be reported in the cities of Manaus and Barcelos, in the state of Amazonas. In the past decade, an outbreak of OROV occurred in the capital of Amazonas [5], as well as in the city of Mazagão, Amapá [6, 7]. In the following years, new OROV epidemics occurred, including in 1988, when outbreaks were reported in Tocantinópolis, state of Tocantins, and Porto Franco, state of Maranhão [8]. In the state of Rondônia, OROV was reported in 1991 in the cities of Ariquemes and Ouro Preto D’Oeste [9]. Since then, OROV has been recorded in several urban centers across the northern and northeastern regions of Brazil [8, 10].

OROV is an enveloped, spherical, and pleomorphic virus with a genome composed of three linear segments of single-stranded, negative-sense RNA [2]. Each RNA segment contains complementary nucleotide sequences that promote the circularization of the viral genome, with a helical nucleocapsid protein that comprises a ribonucleoprotein complex [11]. The tri-segmented genome structure facilitates genetic reassortment, a key mechanism driving viral diversity within this virus group [12–14].

**FIGURE 1:**
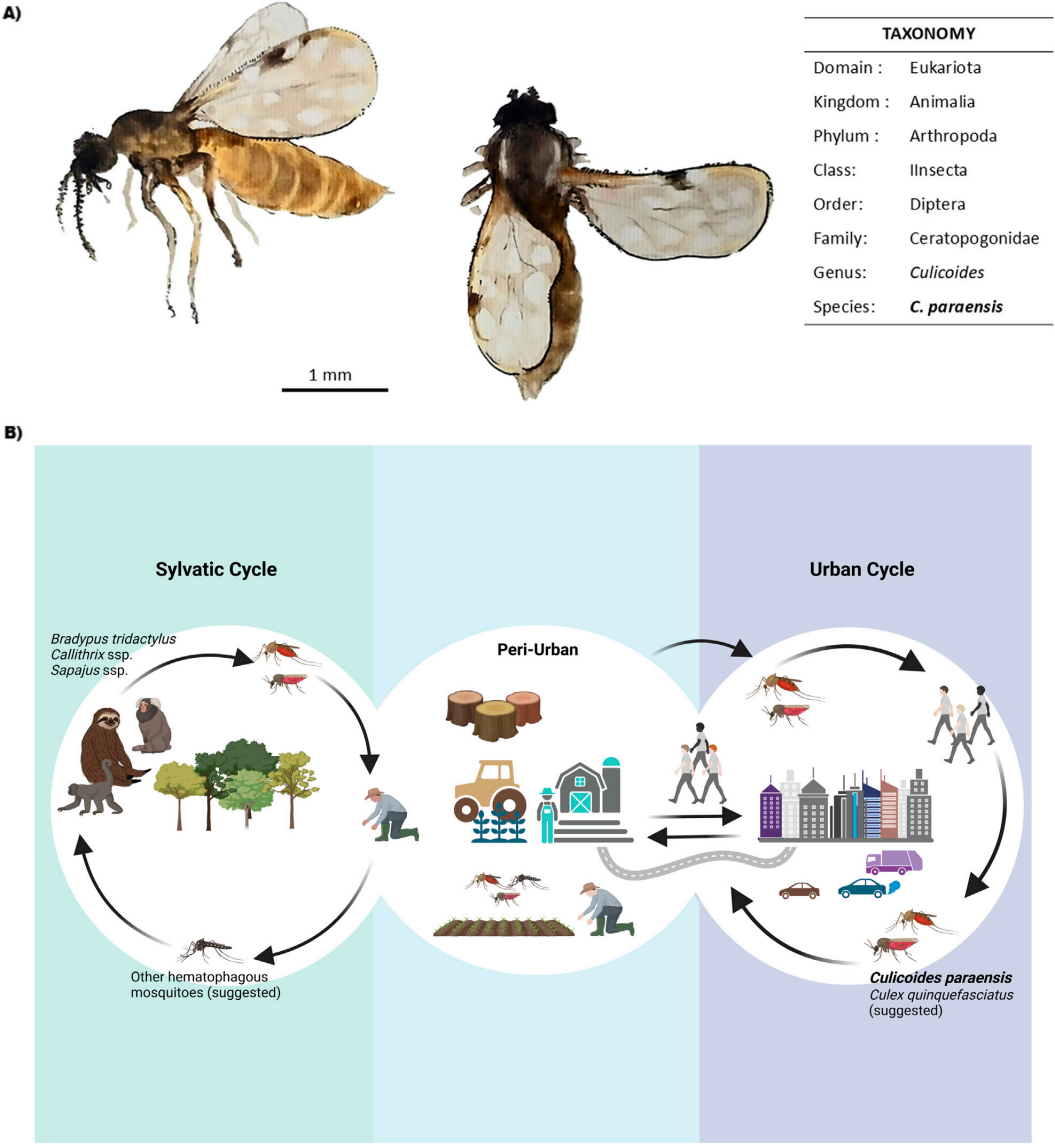
OROV transmission dynamics and main vectors. **(A)** *Culicoides paraensis* general aspect and taxonomic classification. Scale bar = 1 mm. Watercolor Illustration by F. da Fonseca. **(B)** Schematic representation of OROV transmission cycles. The sylvatic cycle involves primates, sloths, and other mammals as hosts, as well as some bird species (not shown). All possible arthropod vectors are not yet well known. The interaction between sylvatic and peri-urban environments primarily occurs due to deforestation and agricultural activities, which favor the transmission of diseases to humans. In the urban cycle, the virus is maintained by vectors such as *Culicoides paraensis* and possibly *Culex quinquefasciatus*, allowing dissemination in densely populated areas. *Original figure created with Biorender*.

OROV is primarily transmitted by biting midges of the species *C. paraensis* [10, 15] (Figure 1A) with reported transmission rates ranging from 25% to 83% [16]. The virus is maintained in nature through two distinct transmission cycles: sylvatic and urban (Figure 1B). In the sylvatic cycle, the virus circulates among wild vertebrate hosts that serve as amplifiers, including non-human primates such *as Callithrix penicillata* [17], *Sapajus apella* [18], and *Alouatta caraya* [19], as well as sloths (*Bradypus tridactylus*) [4] and rodents (*Proechimys spp*.). Antibodies have also been detected in wild birds [20]. Additionally, some studies have identified neutralizing antibodies in domestic animals, including *Canis lupus familiaris* (dogs) and *Bos indicus/taurus* (cattle), in the state of Mato Grosso, Brazil [21]. The duration of viremia in these hosts is critical for the transmission dynamics, as it influences the likelihood of the virus being taken up by biting arthropods during their blood meals. In the urban cycle, *C. paraensis* serves as the primary vector. Humans, acting as accidental hosts, are key in amplifying the virus within urban environments, especially in densely populated regions. The interaction between urban and sylvatic environments creates an ecological bridge, allowing for the virus exchange between the two cycles, thereby contributing to the pathogen’s spread in areas with both natural and anthropogenic factors. The distribution of *C. paraensis* is mainly concentrated in the Amazon basin, but it has also been detected in other tropical areas, facilitating the potential for wider geographical spread of the disease [17–19].

Recent studies have demonstrated the vector competence of other mosquito species for OROV infection in immunocompromised mice, including *Aedes aegypti* and *Aedes albopictus*, in addition to *Culex quinquefasciatus*, suggesting that OROV can be transmitted by more common vector species in the Americas [10, 22]. A recent study analyzed the presence of OROV RNA in seven positive insect individuals, belonging to the species *Cx. quinquefasciatus*, *Limatus durhamii*, and *Aedes albopictus*. Although additional studies on vector competence and capacity are necessary for confirmation, these findings suggest that *Cx. quinquefasciatus* may be a potential primary vector in urban areas [23]. In other environments, the virus has also been isolated from other species, such as *Aedes serratus*, *Coquillettidia venezuelensis*, *Ochlerotatus serratus*, *Mansonia venezuelensis*, and *Aedes aegypti*; nonetheless, the *C. paraensis* remains the main vector [7, 16, 22, 24–27]. Environmental factors such as deforestation, climate change, and other anthropological interferences in the environment accelerate the dispersal of vectors and, consequently, the spread of the disease [28].

Human-to-human transmission has not yet been described in the literature, except for vertical transmission during pregnancy [29–31]. There have been two cases in which the genetic material of the virus was identified in organic tissues of the dead fetus, in the placenta, and in umbilical cord blood [30, 32], in addition to a baby with congenital anomalies who died 47 days after birth [33]. Antibodies to OROV were also found in the serum and cerebrospinal fluid samples from four newborns with neurological malformations. In some cases, fetuses that were infected by OROV during pregnancy presented microcephaly, ventriculomegaly, agenesis of the corpus callosum, and malformation in the joints [25, 32]. Nonetheless, there are still many gaps in the disease transmission cycle and epidemiology that need to be elucidated, such as the probable reservoirs and vectors that promote epizootic events. Indeed, the complex epidemiological features of OROV transmission can be illustrated in a recent report describing the isolation of replication-capable OROV in semen samples from an Italian patient, raising concerns about possible sexual transmission [33].

**FIGURE 2:**
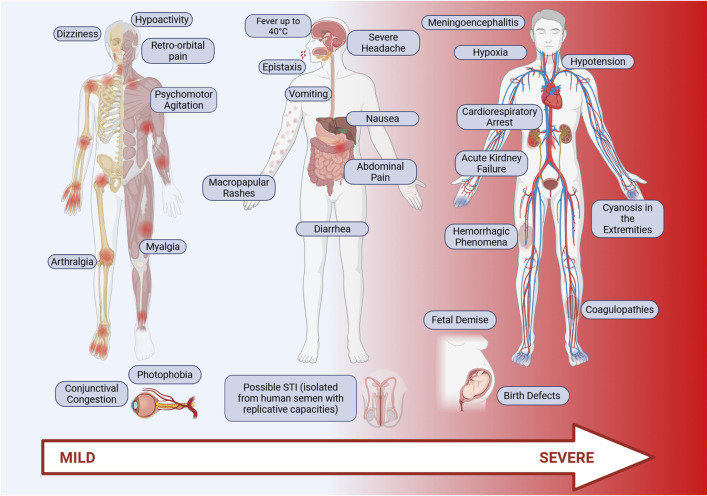
Main symptoms of Oropouche fever. Oropouche fever is characterized by acute clinical symptoms, including high fever (above 39°C), intense headache, retro-orbital pain, myalgia, arthralgia, chills, general malaise, nausea, vomiting, diarrhea, and abdominal pain. Additionally, maculopapular rashes are common, typically starting on the trunk and spreading to the limbs. In some cases, hemorrhagic signs such as epistaxis, gum bleeding, and spontaneous bleeding may occur. Additional symptoms such as photophobia, dizziness, drowsiness, and a burning sensation are frequently observed, especially in more severe infections. Patients with severe forms of the disease may develop coagulopathies, hemorrhagic phenomena, acute kidney failure, and, in extreme cases, death. Infection may also affect the central nervous system, leading to meningitis or encephalitis. *Original figure created with Biorender.*

Oropouche fever is an acute febrile disease with symptoms resembling those of other arboviruses such as Dengue, Zika, Chikungunya, and Mayaro, making clinical diagnosis challenging [5, 34]. The incubation period for OROV is still not well established, but it typically ranges from 3 to 10 days. Following this period, individuals may exhibit a wide range of symptoms, which may vary depending on the virus strain (Figure 2). Common symptoms include high fever (exceeding 39°C, occasionally reaching 40°C), headache, myalgia, arthralgia, chills, malaise, nausea, vomiting, photophobia, retro-orbital pain, diarrhea, abdominal pain, and, in some cases, maculopapular rashes that begin on the torso and spread to the limbs. Hemorrhagic signs, such as spontaneous bleeding, epistaxis, and gum bleeding, have also been reported. With the increase disease spread in 2024, additional symptoms were described, including severe headache, conjunctival congestion, dizziness, drowsiness, severe abdominal pain, anorexia, weakness, and a burning sensation in the body [7, 35]. In some cases, the disease can rapidly progress to more severe manifestations, such as coagulopathies, hemorrhagic phenomena [36, 37], acute kidney failure, and even death [38]. Up to the last quarter of May 2025, 7 deaths were caused by OROV infection in Brazil since the beginning of the current epidemics. Despite the potential for severe complications, the disease is self-limited in most cases, naturally evolving to a complete recovery after 2–7 days of symptoms. However, when the central nervous system is affected, the disease may progress to meningitis or encephalitis, [35, 38, 39]. During the progression of the infection, patients may also present blurred vision, difficulty seeing, hypoactivity, ocular edema, psychomotor agitation, hypotension, hypoxia, cyanosis in the extremities, cold and clammy skin, and, eventually, cardiorespiratory arrest, as observed in a clinical study by Bandeira et al. [36].

## Immunity to OROV and advances in vaccine development and diagnosis

Despite the epidemiological relevance of OROV, the mechanisms of interaction between the virus and the human or animal immune systems remain poorly understood. The innate immune response serves as the first line of defense against OROV, as it does for other infectious agents. The viral evasion mechanisms involved in this response are less specific and share characteristics with the immune escape strategies of arboviruses in general. In response to OROV infection, the adaptive immune system mounts a robust cellular and humoral response. Cytotoxic T lymphocytes (CD8^+^) play a central role in clearing infected cells, while helper T lymphocytes (CD4^+^) contribute by producing cytokines that stimulate B lymphocyte activation and antibody production. The viral envelope glycoproteins GN and GC are critical for viral attachment and entry into host cells and serve as primary targets for neutralizing antibodies [40]. Hematological alterations are also observed during OROV infection, with neutropenia being a common finding, although some patients may exhibit moderate leukocytosis [9].

The currently recognized viral invasion pathways suggest that the blood-brain barrier (BBB) is likely breached during host infection by OROV through a Trojan Horse-like mechanism since human peripheral blood leukocytes were found to have their genome in monocytes, B and T cells, considering the prevalence of neurological manifestations and pathogenesis [41]. In this process, the virus is transported through the bloodstream, hidden within infected phagocytes, and this capacity, after blocking the Interferon pathway, allows the virus to evade immune recognition and reach target organs and tissues, where it can replicate while bypassing barriers such as the BBB in an immunosuppression scenario. The virus had shown infectivity *in vitro* to astrocytes, as wells dysregulating immune innate pathways, and also was found in peripheral blood leukocytes [42, 43], suggesting a major pathway to surpass the barrier and infect neural cells *in vivo*. Nevertheless, a neural invasion pathway may also contribute to OROV pathogenesis, as viral accumulation has been detected within neurons and in *ex vivo* models that preserve brain cytoarchitecture. Notably, infection is associated with the induction of proinflammatory cytokines and neuronal cell death, which may underlie the development of neurological sequelae, potentially of a chronic nature [44]. Studies in suckling mice models have shown viral tropism for neuroprogenitor cells, with glial cells and astrocytes found alongside apoptotic neurons. Moreover, the animals presented splenomegaly and meningitis, although without viral detection in the liver and spleen [45].

The hepatotropic nature of OROV has been previously described [46]. In experimental studies using golden hamsters, high viral titers were detected in the liver, indicating efficient viral replication [487]. Although clinical cases of hepatitis have not been reported in OROV-infected patients, elevated liver enzyme levels have been observed. Notably, in experimental models using mice deficient in interferon regulatory factors, extensive liver damage was documented, supporting the possibility of hepatic involvement. These findings suggest that OROV may exhibit liver tropism, as evidenced by increased serum transaminase levels in patients with Oropouche fever [48].

The activation of the immune system following OROV tissue invasion was evaluated, identifying the key host defense pathways involved in controlling infection and disease progression. Their study demonstrated that OROV pathogenesis and immune responses in primary murine cells occur through RIG-I-like receptor (RLR) signaling, particularly via MDA5, RIG-I, and MAVS. These pathways lead to the activation of type I interferon (IFN-α/β) responses. In knockout models where key regulatory genes such as MAVS, IRF-3, and IRF-7 were suppressed, as well as IFNAR-deficient mice, uncontrolled OROV replication was observed. These animals developed severe hypercytokinemia, liver damage, and high mortality rates, whereas wild-type mice did not develop any signs of severe disease. This finding highlights the critical role of type I IFN responses in restricting viral dissemination. In summary, the induction of type I IFN through MAVS, IRF-3, and IRF-7 is essential for controlling OROV infection in mice [48].

Evasion of the host immune system by *Orthobunyavirus* can occur through the inhibition of the innate antiviral response, particularly by interfering with type I interferon (IFN-α/β) signaling, which is essential for the initial antiviral defense. Viral proteins, including NSs from other bunyaviruses, function as interferon antagonists by inhibiting the transcription of interferon-stimulated genes (ISGs) [49]. Additionally, modulation of autophagy and apoptosis may play a role, as orthobunyaviruses can manipulate cellular processes to facilitate viral replication while preventing early apoptosis in host cells. Another key immune evasion strategy involves the downmodulation of toll-like receptor (TLR) signaling, as the virus can alter viral proteins or modulate host protein patterns to evade TLR recognition. For instance, circumvention of TLR3 signaling, which detects double-stranded RNA, has been described as an evasion strategy by orthobunyaviruses to avoid early immune detection [50]. Understanding the mechanisms of interaction between OROV and the immune system is essential for developing effective control and treatment strategies. Advances in immunological and virological research will provide new insights into mitigating the impact of this emerging pathogen [51].

The lack of vaccines or specific therapies against OROV represents further challenges associated with controlling outbreaks and reducing the impact on public health. The lack of preventive immunizations makes exposed communities entirely dependent on vector control measures, which are often insufficient to contain the spread of the pathogen [52, 53]. In addition, the lack of antiviral therapies limits clinical management, which is based on palliative care to alleviate the symptoms of Oropouche fever. At the same time, rapid and accurate diagnosis plays a critical role in outbreak response and individual patient management [52]. OROV-infected patients present clinical features that are similar to those of other arboviruses, such as dengue, Zika, and chikungunya viruses, making presumptive clinical diagnosis very difficult [54].

The development of vaccines for arboviruses has been a significant challenge *per se*. Genetic instability, high mutation rates, and cross-reactions between some arboviruses are among existing difficulties. In the case of OROV, the segmented nature of its genome further exacerbates the problem as recombination events may increase virus mutation rates as well as the appearance of unforeseen virus variants. In addition, there are currently no animal models that mimic OROV-based human pathophysiology, hindering pre-clinical evaluations of candidate vaccines. These factors represent important gaps for the development of effective immunogens against OROV [9, 55]. Indeed, there have been very few studies on OROV vaccine development. One such study employed the vesicular stomatitis virus as a vector expressing the OROV GPC complex, and immunizations with this experimental vaccine were able to partially protect mice from the appearance of some disease signals after challenges with wild-type OROV [56].

Advances in studies involving other orthobunyaviruses, such as the *Schmallenberg virus* (SBV), may offer valuable insights to be applied to OROV, considering that these viruses share genetic and antigenic characteristics. Inactivated vaccine candidates using different viruses and viral titers were evaluated in cattle and goats, resulting in neutralizing antibody responses and good seroconversion. At least four vaccine candidates employing inactivated viruses significantly reduced viral loads [57]. Another study from the same research group revealed that the use of the SBV Gc glycoprotein as an antigen could confer protective immunity against SBV infection [58]. Furthermore, bioinformatic studies identified a highly immunogenic region within the N protein, which was then used as a vaccine candidate in knockout mice. After immunization with subunit vaccines containing this specific region, mice challenged with virulent SBV showed attenuated clinical signs and reduced viremia. The study also demonstrated that this region of the N protein is conserved among members of the Simbu group, suggesting potential for testing cross-immunity against OROV [59].

The successful management of viral outbreaks has been inherently linked to the availability of effective vaccines; however, two other basic elements in public health approaches are essential to control the disease: i) identification of infected patients through correct diagnosis, and ii) medical support for affected individuals. Indeed, the correct diagnosis of OROV is essential for both individual health management and epidemiological control. The clinical overlap between OROV infections and other febrile illnesses caused by arboviruses, such as Dengue, Zika, and Chikungunya, poses significant diagnostic challenges. Misdiagnosis can result in inappropriate treatments, delayed interventions, and public health inaction. Rapid identification of OROV cases enables the mapping of outbreak zones, assessing viral spread, and the implementation of targeted control strategies, including vector control campaigns and public awareness initiatives [54, 60].

OROV detection typically involves identifying the viral genome in plasma or serum samples from patients in the acute, febrile phase of the disease. A dual-target RT-qPCR assay has been described for the simultaneous detection of Mayaro virus (MAYV) and OROV, focusing on the NSP1 gene of MAYV and the S segment of OROV. This method incorporates customized internal controls using synthetic plasmids containing viral sequences. The protocol, published in 2017, demonstrated high sensitivity and specificity [61], and has been widely adopted by both private and State laboratories in Brazil.

Up to the writing of this review manuscript, no commercial serological tests, such as ELISA and rapid tests, were widely available. Serological tests for detecting IgM and IgG immunoglobulins are carried out using protocols developed in-house, highlighting the fact that the disease has been generally neglected. In 2001, an ELISA using the recombinant nucleocapsid protein (N) as an antigen was described. The protein was produced in a prokaryotic system and tested on samples from patients in Brazil and Peru. The test showed high sensitivity and specificity for detecting Oropouche fever [62]. The development of mouse monoclonal antibodies for the detection of OROV in indirect immunofluorescence (IFA) and immunohistochemistry (IHQ) assays was recently published [63].

Although the development of vaccines and diagnostic tools for OROV presents substantial challenges, recent advances in both areas offer hope for better control and management of this emerging viral threat. The complexity of OROV, with its high mutation rates and clinical similarities to other arboviruses, underscores the urgent need for continued research and innovation. Collaborative efforts and sustained investment in basic and applied research are crucial for developing effective vaccines and diagnostic tools, ultimately enhancing global public health responses to outbreaks of OROV and similar emerging viruses.

## Epidemiology and geographic distribution

In the last 20 years, OROV has been detected throughout South America, including countries such as Peru, Bolivia, Ecuador, Colombia, Venezuela, and French Guiana, besides Brazil [64]. The virus has also been detected again in the Caribbean - Haiti reported an outbreak in 2014 - and sporadic cases were reported in Central America, such as in Panama. Despite all early and late reports, Oropouche fever has been historically underdiagnosed due to its clinical similarity to other arboviral diseases, such as Dengue, Zika, and Chikungunya, making past and present surveillance difficult and current control efforts challenging [65].

In 2011, a novel orthobunyavirus was described in Peru and was retrospectively linked to outbreaks that occurred in 1999. Upon isolation and analysis, it was confirmed that the pathogen, named Iquitos virus, is a recombinant derivative of OROV [14]. Furthermore, in 2017, a study demonstrated the recombination potential between Oropouche and Schmallenberg viruses, both members of the *Peribunyaviridae* family. Although this experiment was conducted under laboratory conditions, it highlights the recombination capability of viruses within this family [66]. To date, four OROV genotypes have been identified: genotype I, found in Trinidad and Brazil; genotype II, identified in Brazil and Peru; genotype III, circulating in Brazil and Panama; and genotype IV, detected in the Amazon region of Brazil [8].

In 2024, the Pan American Health Organization (PAHO) confirmed cases have been reported in eleven countries and one territory in the American continent: Barbados, Bolivia, Brazil, Canada, Colombia, Cuba, Ecuador, United States of America (imported cases), Guyana, Cayman Islands (imported case), Panama, and Peru. Additionally, imported OROV cases have been reported in a few European countries (30 cases in total) [25].

**FIGURE 3:**
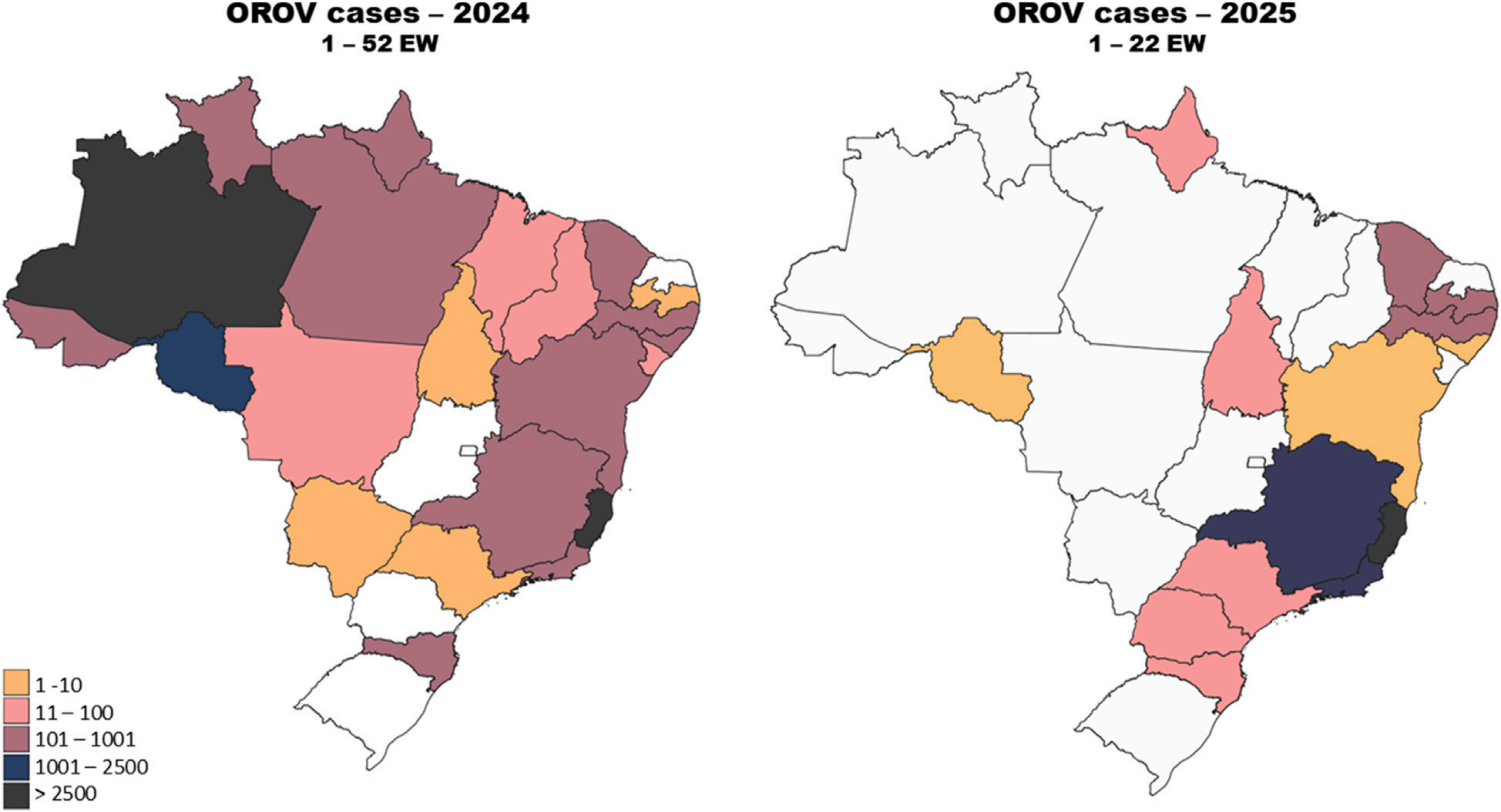
Geographical distribution of Oropouche fever cases in Brazil in 2024 and 2025. Confirmed cases of Oropouche fever in epidemiological week 1–52 of 2024 (left panel). Confirmed cases of Oropouche fever in epidemiological week 1–22 of 2025 (right panel). The Amazon region stands out in 2024, accounting for over 50% of the cases. The spread of the disease beyond the Amazon region reflects the geographical expansion of the disease. In 2025, the state of Espírito Santo accounted for the majority of cases, indicating an early epicenter outside the Amazon region. Epidemiological data on OROV in Brazil were obtained from the Brazilian Ministry of Health [67].

In 2024, Brazil reported cases in 22 out of the country’s 26 states, plus the federal district of Brasilia. This marked a substantial geographic expansion within the Country, implying that outbreaks were no longer limited to the northern region, where the virus is considered endemic. This scenario began in 2023 with three extra-Amazonian cases being reported in the states of Minas Gerais (1 case) and Espírito Santo (2 cases). In the next year, the Amazon basin region accounts for 52.9% of the reported cases, with all of its seven states reporting cases (Table 1). The virus’s spread was noted in several non-endemic areas, including the Southeast, South, and Central-West regions of the Country, further highlighting its increasing reach. Autochthonous transmission was reported in 15 non-Amazonian states [25, 67] (Figure 3; Table 1). Whilst the Amazon and other northern states remain as the epicenters of OROV activity, the emergence of cases in regions such as São Paulo, Rio de Janeiro, and Mato Grosso suggests that the virus’s distribution is shifting, possibly driven by factors like climate change and human movement [67]. In the first 5 months of 2025, Oropouche fever occurrence remained concerningly high, with 11,853 confirmed cases across Brazil up to the end of May. More cases were reported in the first weeks of 2025 than in the same period of 2024, highlighting the rising trend in infections. Cases were recorded in states throughout Brazil [25] (Table 1; Figure 3).

**TABLE 1:** Absolute numbers of OROV cases in Brazil in 2024 (epidemiological week 1–52) and 2025 (epidemiological week 1–22). Epidemiological data on OROV in Brazil were obtained from the Brazilian Ministry of Health and the Pan American Health Organization databases [25, 68].

Region/State	Cases (n)	
	2024 (EW 1-52)	2025 (EW 1-22)
**Northern states**	**5,804**	**109**
Amazonas	3,231	
Rondônia	1,711	7
Acre	276	
Roraima	277	1
Pará	172	1
Amapá	129	87
Tocantins	8	13
**Northeastern states**	**1,517**	**1,837**
Bahia	891	7
Ceará	257	633
Pernambuco	146	643
Alagoas	120	3
Sergipe	34	
Maranhão	33	
Piauí	30	1
Paraíba	6	550
**Southeastern states**	**6,283**	**9,863**
Espírito Santo	5,868	6,271
Minas Gerais	249	1,232
Rio de Janeiro	157	2,302
São Paulo	9	58
**Southern states**	**178**	**43**
Santa Catarina	178	15
Paraná		28
**Center-western states**	**19**	**1**
Mato Grosso	18	
Mato Grosso do Sul	1	1
**Brazil**	**13,801**	**11,853**

## Environmental impacts and OROV expansion

**FIGURE 4:**
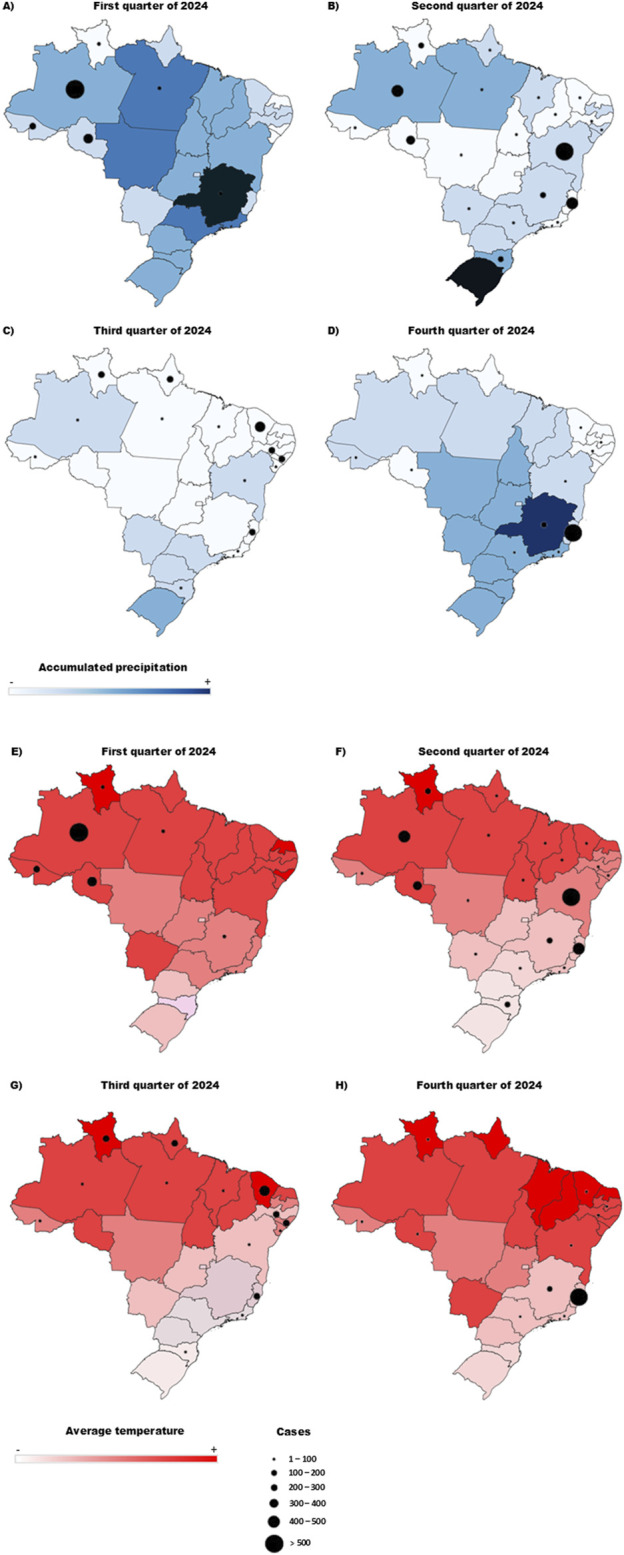
Projection of OROV case numbers across the four-quarters of 2024 concerning temperature and accumulated precipitation. **(A,E)** Relationship between temperature and number of cases in the first trimester/relationship between precipitation accumulation and number of cases in the first trimester. **(B,F)** Relationship between temperature and number of cases in the second trimester/relationship between precipitation accumulation and number of cases in the second trimester. **(C,G)** Relationship between temperature and number of cases in the third trimester/relationship between precipitation accumulation and number of cases in the third trimester. **(D,H)** Relationship between temperature and number of cases in the fourth trimester/relationship between precipitation accumulation and number of cases in the fourth trimester. The color scale determines the temperature gradient **(A–E)** and the accumulated precipitation gradient **(F–G)**, and the size of the circles represents the number of cases.

The spread of the OROV is intricately linked to environmental changes, which alter the dynamics of both vector populations and the virus itself. Climate changes, for instance, has contributed to rising temperatures and irregular rainfall patterns, creating favorable (and sometimes unpredictable) conditions for the proliferation of arthropod vectors. The rainy season in the Amazon region, which lasts from January to June, provides ideal conditions for *C. paraensis* populations to thrive, and during periods of increased rainfall, their populations spike, resulting in a higher risk of transmission. Studies have shown that the increase in precipitation during the rainy season directly correlates with the population numbers of *C. paraensis* and subsequent OROV outbreaks in the region [65]. In Figures 4A–D, the correlation between the number of cases and the average quarterly temperature in 2024 is shown. When evaluating the expansion of the virus to other states (Figures 4B,C), a trend in rising may have contributed to this spread. Conversely, when analyzing the relationship between the number of cases and accumulated precipitation (Figures 4E–H), the peak in cases did not coincide with periods of highest rainfall. However, considering the precipitation from the previous quarter, it is possible that accumulated rainfall created favorable environmental conditions, such as increased humidity and the formation of breeding sites for vectors, which may have influenced the rise in cases in the subsequent quarter.

In addition to climate change, human activities such as deforestation and urbanization have significantly altered local and regional ecology parameters, contributing to the spread of OROV. The loss of native forests and the expansion of urban areas disrupt the habitats of arthropod vectors and reservoir hosts, like primates and sloths. As human settlements encroach on previously forested areas, the proximity between humans, vectors, and reservoirs increases, facilitating the transmission of the virus. In regions like Cusco, Peru, where deforestation has been rampant, outbreaks have been recorded, suggesting that changes in the local ecosystem, such as the loss of vegetation and alterations to animal and insect populations, have contributed to the emergence of OROV [28].

Moreover, the construction of large-scale infrastructure projects, such as dams and roads, has been associated with the spread of OROV. These infrastructure projects often result in environmental changes that promote vector breeding and transference to areas where the insects where previously absent. For example, in the Tucuruí region of Pará State, Brazil, the flooding caused by the construction of a dam created favorable breeding grounds for *Culicoides* mosquitoes, leading to increased occurrence of anti-OROV antibodies in local wildlife, such as in birds and primates [9]. These environmental disruptions, along with changes in local ecosystems, increase the opportunities for the virus to be transmitted between vectors and susceptible hosts [23]. As a result to of regional ecological changes, the virus has now spread from this original confinement in the Amazon basin to neighboring regions and even far states within the Country. The increasing number of outbreaks in the southeastern and southern regions of Brazil, where the virus was previously absent, highlights the combined impact of climate change and human activities on the disease’s distribution. The alteration of ecosystems, the proliferation of vectors, and the movement of people to new areas have all contributed to OROV’s broader geographical spread, challenging public health systems that are not used to managing the disease in non-endemic areas [23].

In a recent study, the complex interaction between ecological and environmental factors, coupled with human mobility, was discussed as a contributing factor to the increase in cases in 2024. The introduction of the virus into large urban centers and its subsequent spread to small inland cities are closely linked to the dynamics of the *C. paraensis* vector, whose opportunistic characteristics and ability to colonize both urban and rural environments favor transmission. Furthermore, the study reveals that agricultural activities, such as banana, cassava, and cocoa crops, provide ideal habitats for mosquito reproduction, creating conditions to the maintenance and spread of the virus. Environmental changes, such as deforestation and the degradation of biomes like the Atlantic Forest and Caatinga, amplify the risk of new outbreaks, as arthropod populations tend to search for new environments to survive, highlighting the importance of continuous surveillance. In addition to ecological and environmental factors, genomic studies have revealed that the current OROV epidemic may have been driven by the emergence of a novel reassortant lineage, designated OROVBR-2015-2024, which combines genomic segments from distinct geographical origins. This lineage presents synapomorphic non-synonymous mutations in both the L segment, which encodes the RNA-dependent RNA polymerase, and the M segment, which encodes the viral envelope glycoproteins. Notably, mutations such as I957V in the Amazonian AM-I clade and I958T in the non-Amazonian SC-2 clade are located in the M segment and may play a role in modulating viral entry or immune evasion. These genetic alterations are particularly relevant, as the M segment directly influences viral tropism and host cell interactions, potentially affecting replication kinetics and transmission dynamics. Altogether, these findings suggest that viral adaptation may have contributed to increased transmissibility or fitness of OROV outside the Amazon region [60].

Furthermore, climate models suggest that the continued warming of the planet and changes in precipitation patterns will further affect the distribution of OROV vectors. As temperatures rise and rainfall becomes more erratic, new regions may become suitable for *Culicoides* vectors to proliferate, creating new hotspots for OROV outbreaks. This phenomenon is particularly concerning because it may introduce the virus into regions with less experience in managing vector-borne diseases, making them more vulnerable to outbreaks.

## Discussion

Oropouche fever remains a significant public health concern in tropical Latin America. Although originally confined to the Amazon basin, OROV has recently expanded into southeastern and southern Brazil, reflecting changes in its epidemiological dynamics. This expansion is driven by increased human population mobility, urban growth, and environmental factors such as rising temperatures and altered rainfall patterns that favor vector proliferation. Addressing OROV transmission requires an integrated approach that considers both ecological pressures and public health interventions.

The pathogenesis of OROV is still not fully elucidated, particularly in terms of viral-host immune interactions. The absence of specific antiviral treatments and licensed vaccines continues to hinder effective disease control. While diagnostic technologies have improved, early detection and surveillance remain limited—especially in endemic and resource-constrained areas. Strengthening diagnostic capacity is a short-term priority to enable rapid response to emerging outbreaks.

Environmental changes, such as deforestation and large-scale infrastructure development, have disrupted vector habitats and facilitated viral spread beyond traditional endemic zones. However, repeated references to these drivers across studies highlight the urgent need for long-term strategies that address root ecological causes. Coordinated public policies that align health surveillance with environmental protection are essential. In the long term, controlling OROV will require not only continued genomic and ecological monitoring but also investment in vaccine research and sustainable land-use practices to mitigate vector expansion. From the legal point of view, it is important to mention that Brazil possesses a robust legal framework for environmental protection, which extends beyond the Amazon - its most internationally recognized biome - to include all other, lesser-known ecosystems within the national territory. Moreover, Brazil is a signatory to all editions of the Conference of the Parties (COP). Notably, COP30 will be held in Belém, the most influential and significant Brazilian capital in the Amazon region. The conference is scheduled to take place in November 2025 and is already being referred to as the ‘Amazon COP’.

The current regional OROV outbreak must be addressed within a One Health framework, recognizing the interconnectedness of human, animal, and environmental health. Key aspects warranting further investigation include the role of environmental and anthropogenic factors in peri-urban areas, the presence of secondary vectors capable of supporting OROV replication, and the occurrence of imported cases in regions where Culicoides species of the same genus are present. Additionally, exploring whether the OROVBR-2015-2024 variant exhibits altered vector tropism, hepatocyte affinity, or neurotropism in extra-Amazonian contexts could reveal important pathophysiological insights. A deeper understanding of virus-host interactions—both *in vivo* and *ex vivo*, including apoptosis pathways—may help uncover critical knowledge gaps. Such efforts are essential for improving diagnostic capabilities and informing the development of effective antivirals, vaccines, and therapeutic strategies to mitigate the public health impact of Oropouche virus infections [68].
